# Acknowledgement

**Published:** 2024

We are extremely grateful to the following members of the medical profession from Pakistan and Overseas for sparing their precious time to review the manuscript published in the Issue of January – February (Part-I) 2024.

**Table:** 

*Reviewer’s Name*	*(Manuscript* *Reviewed)*	*Reviewer’s Name*	*(Manuscript* *Reviewed)*	*Reviewer’s Name*	*(Manuscript* *Reviewed)*
Adeela Shahid, ***Pakistan***		Mohammad Kamran, ***Pakistan***		Sultan Abdulwadoud Alshoabi, ***Saudi Arabia***	
Afshan Shahid, ***Pakistan***		Mohammad Moazzam, ***Pakistan***		Sumera Ehsan, ***Pakistan***	
Ahmad Muhammad, ***Qatar***		Maria Sarwar, ***UK***		Syed Zulfiqar Ali Shah, ***Pakistan***	
Alimova Sekina, ***Russia***		Maryam Azfar, ***Pakistan***		Syeda Amina Ahmad, ***Pakistan***	
Ammad Asghar, ***Pakistan***		Masood Jawaid, ***Pakistan***		Tariq Mahmood Tahir, ***Pakistan***	
Anita Haroon, ***Pakistan***		Mian Ayaz ul Haq, ***Pakistan***		Tariq Wahab Khanzada, ***Oman***	
Anjum Jalal, ***Pakistan***		Mubashir Rasheed, ***Pakistan***		Tayyaba Gul Malik, ***Pakistan***	
Ayesha Ayub, ***Pakistan***		Mujeed-ur-Rehman Dawar, ***Pakistan***		Ting Zhang, ***China***	
Chen-li Zhang, ***China***		Musarrat Riaz, ***Pakistan***	7	Wang Jinfei, ***China***	
Ci Liu, ***China***		Nasir Khokar, ***Pakistan***		Waris Qidwai, ***Pakistan***	
Dong Xing, ***China***		Nazish Imran, ***Pakistan***		Xiao-min Meng, ***China***	
Fahad Azam, ***Pakistan***		Noor-i-Kiran Naeem, ***Pakistan***		Ya-bin Liu, ***China***	
Fazal Akbar, ***Pakistan***		Riffat Farooqui, ***Pakistan***		Younis Abdelwahab Skaik, ***Germany***	
Ghulam Sarwar Pirkani, ***Pakistan***		Rubina Naqvi, ***Pakistan***		Ziauddin Shaikh, ***UK***	
Haris Majid, ***Pakistan***		Rufina Soomro, ***Pakistan***		Zubia Masood, ***Pakistan***	
Huma Majeed Khan, ***Pakistan***		Syed Fawad Mashhadi, ***Pakistan***			
Humaira Zafar, ***Pakistan***	4	Syed Hamid Habib, ***Pakistan***			
Huseyin Selvi, ***Turkey***		Syed Hussain Raza Zaidi, ***Pakistan***			
Ikramullah, ***Pakistan***		Syed Mamun Mahmud, ***UAE***			
Javeria Nafis Khan, ***Pakistan***		Saba Majeed, ***Pakistan***			
Javeria Usman, ***Pakistan***		Saba Sohail, ***Pakistan***			
Jun Wu, ***China***		Sabri Selcuk Atamanalp, ***Turkey***			
Khemchand N. Moorani, ***Pakistan***		Sadaf Arooj, ***Pakistan***			
Laima Alam, ***Pakistan***		Sajid Majid, ***Pakistan***			
Laima Yusuf, ***Pakistan***		Sajida Qureshi, ***Pakistan***			
Ling-ling Li, ***China***		Salman Jamil, ***Pakistan***			
Lin-lin Feng, ***China***		Shahid Sarwar, ***Pakistan***			
Linlin Wu, ***China***		Shamila Ijaz Munir, ***Pakistan***			
Mohammad Yasir Khan, ***Pakistan***	2	Shireen Jawed, ***Pakistan***			
Mohammad Ashraf Ganatra		Sidra Jazil Faruqi, ***Pakistan***			
Mohammad Aslam, ***Pakistan***	2	Sinan Guzel, ***Turkey***			
Muhammad Fuad Bangash, ***USA***					

